# Long non-coding RNA CASC2 suppresses epithelial-mesenchymal transition of hepatocellular carcinoma cells through CASC2/miR-367/FBXW7 axis

**DOI:** 10.1186/s12943-017-0702-z

**Published:** 2017-07-17

**Authors:** Yufeng Wang, Zhikui Liu, Bowen Yao, Qing Li, Liang Wang, Cong Wang, Changwei Dou, Meng Xu, Qingguang Liu, Kangsheng Tu

**Affiliations:** Department of Hepatobiliary Surgery, The First Affiliated Hospital of Xi’an Jiaotong University, Xi’an, Shaanxi Province 710061 China

**Keywords:** Long non-coding RNA, Hepatocellular carcinoma, Epithelial-mesenchymal transition, CASC2, miR-367, FBXW7

## Abstract

**Background:**

Recently, it has been reported that long non-coding RNA (lncRNA) cancer susceptibility candidate 2 (CASC2), a novel tumor suppressor, participates in regulating the carcinogenesis and suppresses tumor progression by sponging microRNAs (miRNAs). However, the expression and function of CASC2 in hepatocellular carcinoma (HCC) remain unclear.

**Methods:**

The expression of CASC2 and miR-367 in HCC specimens and cell lines were detected by real-time PCR. Western blotting and immunohistochemistry were carried out for detection of epithelial-to-mesenchymal transition (EMT) markers in HCC. Transwell assays were used to determine migration and invasion of HCC cells. A mouse model for lung metastasis was established to evaluated HCC metastasis in vivo. The correlation among CASC2, miR-367 and F-box and WD repeat domain containing 7 (FBXW7) were disclosed by a dual-luciferase reporter assay, RIP assay and biotin pull-down assay.

**Results:**

Here, CASC2 expression was significantly downregulated in HCC tissues, especially in aggressive and recurrent cases. In accordance, CASC2 underexpression was observed in HCC cell lines compared to LO2. In vitro and in vivo experiments revealed that CASC2 inhibited migration and invasion of HCC cells. Additionally, CASC2 repressed EMT process of HCC cells. Further studies demonstrated that CASC2 could function as a competing endogenous RNA (ceRNA) by sponging miR-367 in HCC cells. Functionally, gain- and loss-of-function studies showed that miR-367 promoted migration, invasion and EMT progression of HCC cells. Moreover, further investigations disclosed that FBXW7 was a downstream target of miR-367 and CASC2 prohibited EMT progression and subsequently exerted its anti-metastatic effects via CASC2/miR-367/FBXW7 axis in HCC cells. Clinically, CASC2 underexpression and miR-367 overexpression were closely correlated with the metastasis-associated clinicopathologic features. Notably, CASC2 low-expressing and miR-367 high-expressing HCC patients showed the poorest clinical outcome.

**Conclusions:**

Overall, we conclude that the CASC2/miR-367/FBXW7 axis may be a ponderable and promising therapeutic target for HCC.

**Electronic supplementary material:**

The online version of this article (doi:10.1186/s12943-017-0702-z) contains supplementary material, which is available to authorized users.

## Background

Hepatocellular carcinoma (HCC) is one of the main causes for cancer-associated deaths worldwide [1, 2]. Although remarkable improvements of therapeutic strategy for HCC have been made, surgery is still the best treatment option [1]. One of the main obstacles in HCC treatment is the high incidence of cancer metastasis, which is also the main reason for poor prognosis [1]. Thus, in order to promote the research development of therapeutic targets for HCC, expounding the possible molecular mechanisms involved in malignant biological behaviors of cancer cells is especially important.

Previous studies confirm that epithelial-to-mesenchymal transition (EMT), contributing to the cancer invasion and metastasis cascade, has been identified as a critical process in the progression of cancers including HCC [3]. Long non-coding RNAs (lncRNAs) and microRNAs (miRNAs) are two kinds of non-coding RNAs, which are not able to encode any protein [4]. The existing researches have showed that the abnormally expressed lncRNAs, miRNAs and proteins regulate the EMT progression of HCC cells via their interactions [5–7]. They are recognized as valuable and sanguine therapeutic targets to withstand the metastasis of HCC. For example, lncRNA ATB, a regulator of transforming growth factor-β (TGF-β) signaling, could competitively bind to the miR-200 family and accordingly increased the expressions of zinc finger E-box binding homeobox 1 (ZEB1) and ZEB2, and then promoted the metastasis, invasion and EMT progression in HCC [7]. Moreover, lncRNA HULC regulates HCC cell preternatural lipid metabolism via the HULC/miRNA-9/RXRA axis [8].

LncRNA cancer susceptibility candidate 2 (CASC2), a novel tumor suppressor, modulates cell migration, invasion, proliferation, apoptosis and tumor growth in multiple human cancers, such as renal cell carcinoma, gastric cancer, non-small cell lung cancer, endometrial cancer, colorectal cancer and glioma [9–12]. For instance, CASC2 could restrain glioma cell invasion, migration and proliferation by negatively regulating miR-21 expression [10]. In gastric cancer, CASC2 might serve as a tumor suppressor that suppressed cell proliferation by inactivation of mitogen-activated protein kinase (MAPK) pathway [9]. However, whether these functions of CASC2 exist in HCC development remains unclear. Besides, it has been suggested that miR-367 acts as an oncogene to promote the invasive, migratory and proliferative abilities of HCC cells [13]. Otherwise, miR-367 promotes the cell migration and invasion via targeting F-box and WD repeat domain containing 7 (FBXW7, [14]). Additionally, it has been reported that FBXW7 suppresses migration, invasion, and EMT progression of HCC cells [15]. However, whether miR-367 could promote EMT progression of HCC cells via targeting FBXW7 remains unknown.

Here, this study demonstrated that CASC2 was dramatically underexpressed and could suppress migration, invasion and EMT process of HCC cells. On the other hand, miR-367 was overexpressed and could promote EMT progression and played an pro-metastatic role in HCC. Moreover, CASC2 was recognized as a competing endogenous RNA (ceRNA) for miR-367 and could exert its anti-metastatic effects on cell migration, invasion and EMT progression through CASC2/miR-367/FBXW7 axis, which might inject some new vitalities into the development therapeutic targets for HCC.

## Methods

### Clinical specimens

Seventy-five HCC tissues and adjacent normal tissues were collected from patients, who underwent hepatectomy in the Department of Hepatobiliary Surgery, the First Affiliated Hospital of Xi’an Jiaotong University during January 2009 to December 2011. All patients did not receive any embolotherapy or chemotherapy before surgical operation and were pathologically diagnosed post-operation.

### Cell culture and transfection

Six HCC cell lines (MHCC-97L, Hep-3B, HepG2, Huh7, SMMC-7721 and MHCC-97H) and the human immortalized normal hepatocyte cell line (LO2) were cultured in DMEM (Gibco, USA) with 10% fetal bovine serum (FBS, Gibco), 100 μg/mL streptomycin and 100 U/mL penicillin (Sigma, USA). All of the cells were maintained in an incubator (5% CO_2_, 37 °C). According to the product description, cell transfection were performed using Lipofectamine 2000 Reagent (Invitrogen Life Technologies, USA). Scrambled siRNA (siControl) and CASC2 siRNAs (siCASC2#1 and siCASC2#2), as well as pcDNA3.1-Control (pcDNA/Control) and pcDNA3.1-CASC2 (pcDNA/CASC2) were purchased from Invitrogen (USA). MiR-367 inhibitors (anti-miR-367), inhibitors negative control (NC), miR-367 mimics (miR-367) and mimics negative control (NC) were purchased from Genecopoeia (Guangzhou, China). FBXW7 Human cDNA ORF Clone and Control were purchased from OriGene (OriGene Technologies, Inc., USA).

### Dual luciferase reporter assay

Bioinformatics tools (microRNA.org, Starbasev2.0, miRcode et al.) were applied to analyze the miR-367 binding sites on CASC2 gene as well as the FBWX7 binding sites on miR-367 gene. Then a dual luciferase reporter assay was conducted according to the protocols described in our previous study [16]. Subsequently, the luciferase reporter assay system (Promega, Madison, WI, USA) was applied to examine the luciferase activity. All of the above experiments were conducted repeatedly for three times.

### RNA immunoprecipitation (RIP) assay

The EZ-Magna RIP Kit (Millipore, USA) was applied to conduct the RIP assay according to the product specification. Firstly, cells were collected and lysed in complete RIP lysis buffer. Then, the cell extract was incubated with RIP buffer containing magnetic beads conjugated to a human anti-Ago2 antibody (Millipore, USA). Samples were incubated with proteinase K with shaking to digest proteins and the immunoprecipitated RNA was isolated. Subsequently, the NanoDrop spectrophotometer was used to measure the concentration of RNA, and the purified RNA was subjected to real-time PCR analysis.

### Biotin pull-down assay

The DNA fragment with the full length CASC2 sequence or negative control sequence was PCR amplified using a T7-containing primer and then cloned into GV394 (Genechem, Shanghai, China). The resultant plasmids DNAs were linearized using restriction enzyme XhoI. Biotin-labeled RNAs were reversely transcribed using Biotin RNA Labeling Mix (Roche, USA) and T7 RNA polymerase (Takara Biomedical Technology). The products were treated with RNase-free DNase I (Roche, USA) and purified with the RNeasy Mini Kit (Qiagen, USA) and RNA were extracted for real-time PCR.

### Isolation of RNA and real-time PCR analysis

Based on the manufacturer’s protocol of Trizol (Invitrogen, Carlsbad, CA, USA), total RNA from cells and tissues were isolated.Then the cDNA was obtained according to the protocols described in our previous study [16], and RNA levels were measured by real-time PCR analysis. Primers for CASC2, miR-367 and FBXW7 were purchased from Genecopoeia (Guangzhou, China). Then the results were calculated applying the 2^-ΔΔCt^ method.

### Western blot

Cells were harvested and then lysed via radioimmunoprecipitation assay buffer (RIPA buffer, Thermo Fisher Scientific, MA,USA) to obtain proteins. Subsequently, proteins were separated by SDS–PAGE and transferred to PVDF membranes. Then the above PVDF membranes were incubated with corresponding primary antibodies. E-cadherin (1:1000, Cell Signaling, MA, USA), Vimentin (1:500, Santa Cruz, CA, USA), FBXW7 (1:1000, Abcam, MA, USA) and β-actin (1:500, Santa Cruz, CA, USA). Then the membranes were incubated with HRP-conjugated secondary antibody for 2 h at room temperature (ZSGB-BIO, China). Detection was performed by enhanced chemiluminescence kit (Amersham, Little Chalfont, UK).

### Transwell assay

The upper chambers of transwell inserts (8 μM pore-sized, Nalge Nunc Intl., NY, USA) were coated with matrigel (1:8, BD, NJ, USA) for invasion assay, while migration assay without matrigel. Then the subsequent experiments were conducted according to the protocols described in our previous study [16]. After 24 h, cells were fixed using 4% paraformaldehyde, and permeabilized with methanol. After removing these cells still in the upper chambers, the cells in lower chambers were stained with 0.3% crystal violet dye and washed by PBS. The images were obtained and cells were counted using a light microscope (×100).

### Immunohistochemistry staining

The experiments was conducted according to the protocols described in our previous study [16]. The sections were dewaxed, dehydrated, and rehydrated. Then, citrate buffer was used for retrieve antigen, and hydrogen peroxide (3.0%) was used for blocking the endogenous peroxidase activity. After being blocked by 10% goat plasma, the primary antibodies, including E-cadherin (1:400, Cell Signaling) and Vimentin (1:200, Santa Cruz) were added to the sections and incubated at 4 °C overnight. The biotinylated secondary antibodies (Goldenbridge, Zhongshan, China) applied for detecting the primary antibodies. Counterstained was performed using hematoxylin.

### Lung metastasis in vivo

In order to assess the effect of CASC2 on metastatic ability of HCC cells in vivo, the lentivirus-based system was applied to establish CASC2 stably overexpressing MHCC-97H cells and CASC2 down-regulating Hep-3B cells. Then, these MHCC-97H cells (1 × 106) and Hep-3B cells (1 × 106) were injected into the tail vein of nude mice. Eight weeks later, the lungs of nude mice were gathered and fixed with dampen formaldehyde solution. Then paraffin-embedded, routine hematoxylin-eosin (H&E) staining and morphology observed by microscope were performed to evaluate the lung metastases. The protocols for these animal experiments were approved by the Ethics Review Committee of Xi’an Jiaotong University.

### Statistical analysis

Data were represented as mean ± SD and analyzed by the softwares, SPSS Version 22.0 (SPSS, Chicago, USA) and Graphpad Prism 6.0 (CA, USA). The statistical approaches mainly included a two-tailed Student’s t test, ANOVA, a Kaplan–Meier plot, Log-rank test, Pearson chi-squared test and Pearson’s correlation analysis. Difference with *P* < 0.05 was regard to be statistically significance.

## Results

### The downregulation of CASC2 in HCC tissues and cell lines

**Fig. 1:**
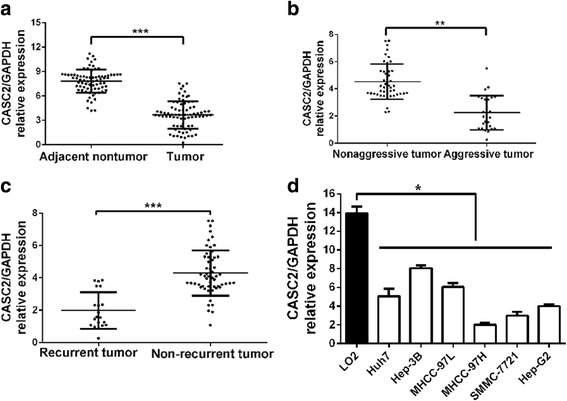
CASC2 expression was downregulated in HCC. **a** The expression of CASC2 in tumor tissues was significantly lower than that in adjacent nontumor tissues. **b** The expression of CASC2 in aggressive tumor tissues was significantly lower than that in nonaggressive cases. **c** The expression of CASC2 in HCC tissues with recurrence was significantly lower than that in HCC tissues without recurrence. **d** CASC2 expression was downregulated in HCC cell lines compared to normal hepatocyte LO2. *P < 0.05, **P < 0.01, ***P < 0.001

Firstly, real-time PCR was applied to measure CASC2 expression in 75 paired HCC tissues and adjacent non-tumor tissues. The results revealed that CASC2 expression in HCC tissues was dramatically decreased, compared with adjacent non-tumor tissues (*P* < 0.001, Fig. 1a). Furthermore, CASC2 expression was markedly downregulated in aggressive HCC tissues compared with non-aggressive HCCs (*P* < 0.01, Fig. 1b). CASC2 expression was notably decreased in HCC tissues with recurrence compared to those without recurrence (*P* < 0.001, Fig. 1c). Meanwhile, The data from R2: Genomics Analysis and Visualization Platform (http://r2.amc.nl) including GEO and TCGA database showed that the expression of CASC2 was significantly downregulated in HCC tissues compared to normal liver tissues (*P* < 0.05, respectively, Additional file 1: Fig. S1), which were consistent with our results. Additionally, our data revealed that significantly underexpressed CASC2 could be observed in all the six HCC cell lines, compared to LO2 cells (*P* < 0.05, respectively, Fig. 1d). Hep-3B cells had the highest expression of CASC2 and MHCC-97H cells showed the lowest level (Fig. 1d), thus these two cell lines were used for loss- and gain-of-function experiments. In conclusion, these data suggested that CASC2 expression was markedly decreased in HCC.

### CASC2 suppresses migration, invasion and EMT of HCC cells

**Fig. 2:**
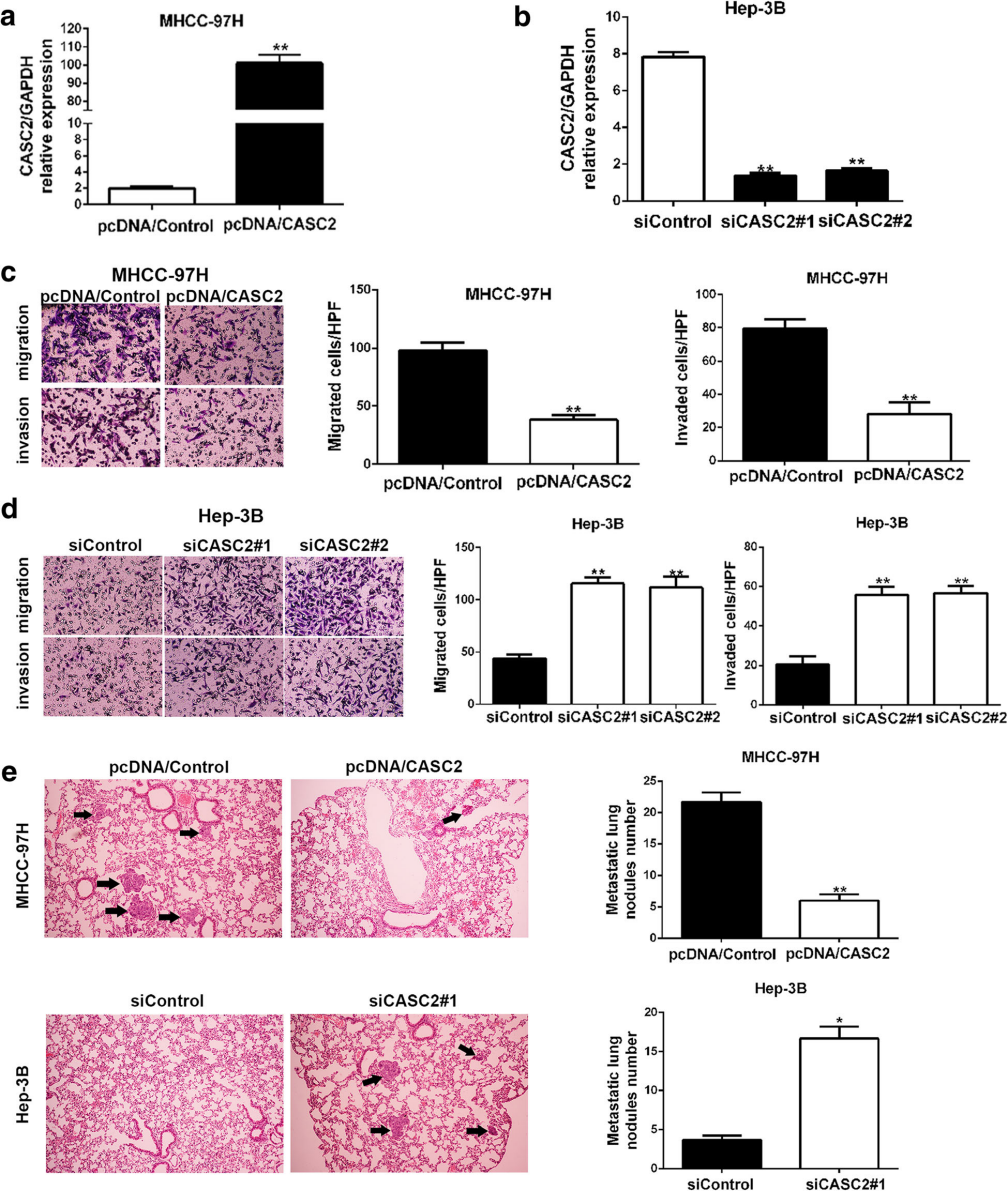
CASC2 inhibited migration and invasion of HCC cells both in vitro and in vivo. **a** pcDNA/CASC2 could significantly increase CASC2 expression in MHCC-97H cells. **b** CASC2-siRNAs could significantly decrease CASC2 expression in Hep-3B cells. **c** Transwell assays showed that CASC2 restoration decreased migration and invasion abilities of MHCC-97H cells. **d** CASC2 knockdown facilitated migration and invasion of Hep-3B cells. **e** Representative pictures showed lung metastasis of HCC in mouse model (magnification: ×100). *Black arrow* showed the position of lung metastasis (*left panels*). Quantitative analysis was used to compare the lung metastasis nodules between these two groups (*right panels*). *P < 0.05, **P < 0.01

Next, we attempted to investigate the functional effects of CASC2 on HCC cells. Firstly, MHCC-97H cells were transfected with pcDNA/CASC2, while Hep-3B cells were transfected with CASC2 siRNAs (siCASC2#1 and siCASCC2#2). Real-time PCR analysis revealed that the expression of CASC2 was significantly modulated by pcDNA/CASC2 and CASC2-siRNAs in MHCC-97H and Hep-3B cells (*P* < 0.01, respectively Fig. 2a and b). Functionally, Transwell assays demonstrated that overexpressed CASC2 could significantly diminish the migration and invasion abilities of MHCC-97H cells (*P* < 0.01, respectively Fig. 2c). On the other hand, CASC2 silencing significantly potentiated the cell migration and invasion of Hep-3B cells (*P* < 0.01, respectively Fig. 2d). Furthermore, we tried to confirm the functional effects of CASC2 on HCC cells in vivo. The results revealed that CASC2 stably overexpressing MHCC-97H cells appeared lower probability of lung metastasis, while CASC2 underexpressing Hep-3B cells presented much more lung metastasis compared to control group (*P* < 0.05, respectively Fig. 2e). These data indicated that CASC2 could repress migration and invasion of HCC cells both in vitro and in vivo.

**Fig. 3:**
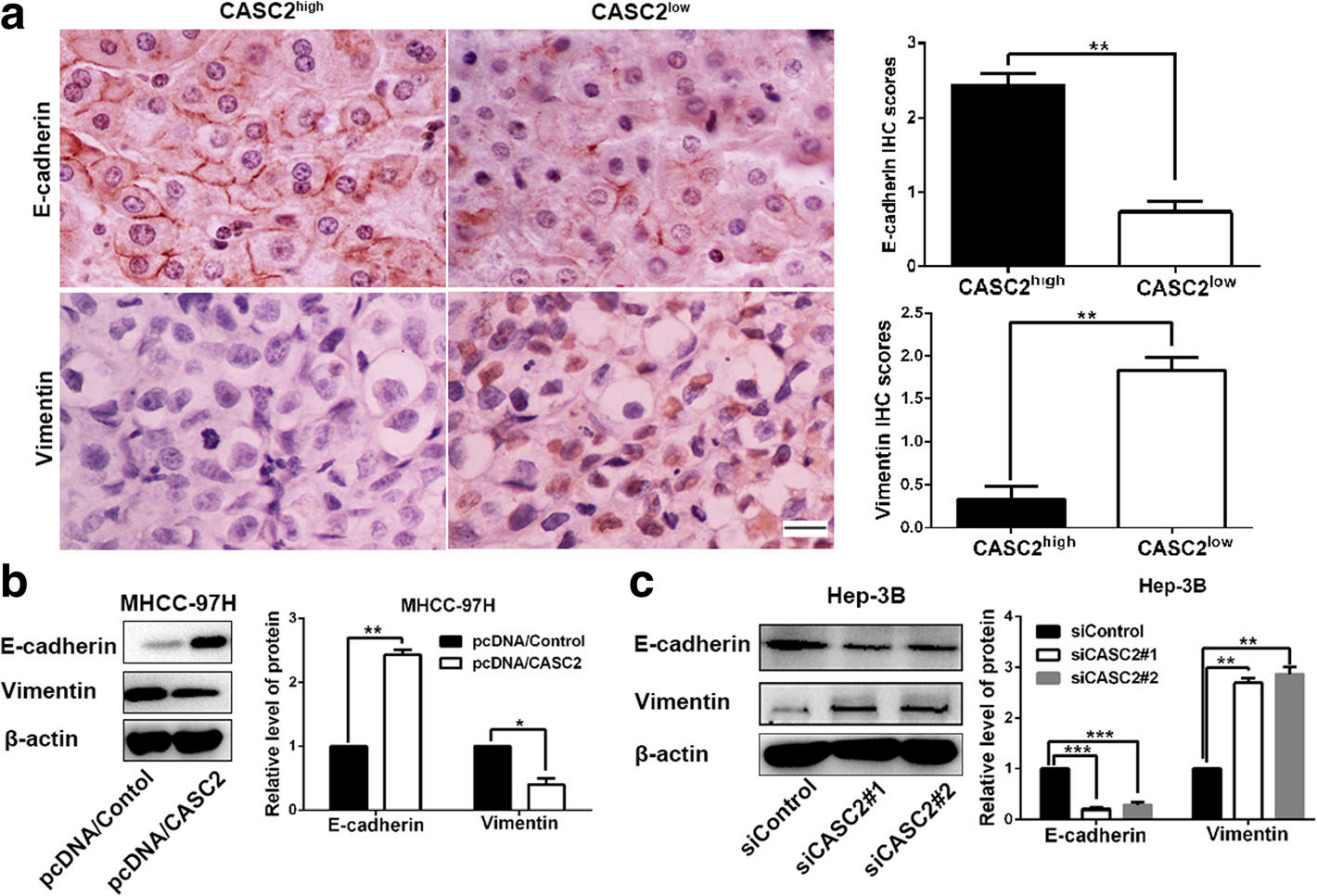
CASC2 suppressed the EMT progression of HCC cells. **a** Immunohistochemistry analysis (magnification: ×400; Scale bar:100 μm) showed that HCC tissues in high CASC2 group presented higher E-cadherin expression and lower Vimentin expression compared to those in low CASC2 group. **b** CASC2 restoration significantly increased E-cadherin expression and decreased Vimentin expression in MHCC-97H cells. **c** CASC2 silencing could significantly decreased E-cadherin expression and increased Vimentin expression in Hep-3B cells. *P < 0.05, **P < 0.01, ***P < 0.001

EMT progression is of great importance for migration and invasion of HCC cells. Then, we attempted to explore whether CASC2 could exert any influence on EMT progression of HCC cells. Immunohistochemical staining analysis was conducted in 75 HCC samples to explore the associations between CASC2 and EMT markers (Vimentin and E-cadherin). Subgroups (CASC2 low/high) were divided according to the cutoff values of CASC2, which were defined as the median of the cohort. The results indicated that E-cadherin expression was significantly decreased while Vimentin expression was obviously increased in low CASC2 group compared to high CASC2 group (*P* < 0.01, respectively Fig. 3a). Consistently, Western blot results revealed that CASC2 overexpression could significantly elevate the expression of E-cadherin and repress the expression of Vimentin in MHCC-97H cells (*P* < 0.05, respectively Fig. 3b). CASC2 silencing reduced E-cadherin expression and increased Vimentin in Hep-3B cells (*P* < 0.01, respectively Fig. 3c). These results demonstrated that CASC2 inversely regulated EMT progression in HCC cells.

### MiR-367 was a target of CASC2

**Fig. 4:**
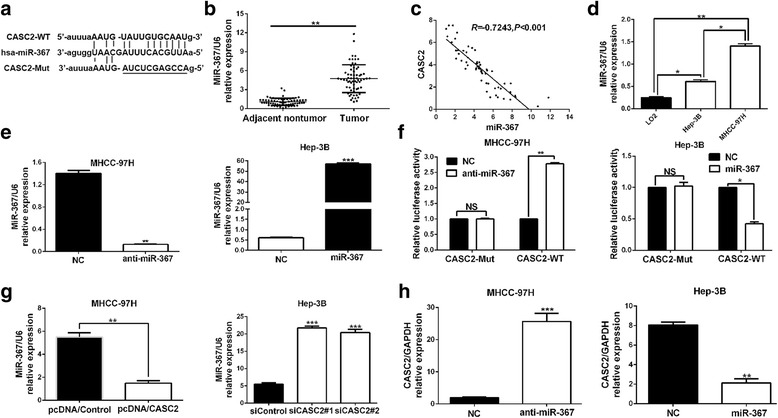
miR-367 was a target of CASC2 in HCC. a Bioinformatics analysis showed that miR-367 could directly target 3′-UTR of CASC2-wild type (WT). CASC2-mutant (Mut) means mutation of binding sites in the 3′-UTR of CASC2. **b** The expression of miR-367 in tumor tissues was significantly higher than that in adjacent nontumor tissues. **c** Pearson correlation analysis revealed that an obvious negative association between miR-367 and CASC2 expression in HCC tissues. **d** The expression of miR-367 in LO2, MHCC-97H and Hep-3B cells as detected by real time-PCR. **e** miR-367 mimics or inhibitors could significantly modulate the expression of miR-367 in HCC cells. **f** Dual luciferase reporter assays showed that miR-367 could negatively regulate the luciferase activity of CASC2-WT, rather than CASC2-Mut. **g** Real-time PCR showed that CASC2 could negatively regulate miR-367 expression in HCC cells. **h** miR-367 inversely regulate CASC2 expression in HCC cells. *P < 0.05, **P < 0.01, ***P < 0.001

To explore the potential mechanisms of CASC2 functions in HCC cells, several bioinformatics tools (microRNA.org and miRBase) were employed to analyze the potential targets of CASC2. Bioinformatics tools analysis revealed the potential binding site for miR-367 on CASC2 gene (Fig. 4a). And the data of real-time PCR showed that the expression of miR-367 was markedly elevated in HCC tissues (*P* < 0.01, Fig. 4b). Pearson correlation analysis revealed that a negative association between miR-367 and CASC2 expression was observed in HCC tissues (*r* = −0.7243, *P* < 0.001, Fig. 4c). In addition, we detected the expression of miR-367 in LO2, MHCC-97H and Hep-3B cells. The data indicated that both MHCC-97H and Hep-3B cells showed a higher miR-367 expression compared to LO2 cells (*P* < 0.05, respectively Fig. 4d). Meanwhile, MHCC-97H had the highest expression of miR-367 (*P* < 0.05, Fig. 4d). Next, MHCC-97H and Hep-3B cells were transfected miR-367 inhibitors and mimics, respectively. Then, miR-367 overexpression and knockdown were confirmed by real-time PCR (*P* < 0.01, respectively Fig. 4e). The luciferase reporter assays manifested that miR-367 significantly suppressed the luciferase activity that carried wild type (WT) but not mutant (Mut) 3′-UTR of CASC2 (*P* < 0.05, respectively Fig. 4f). In addition, it has been well identified that miRNAs exert its function via binding to Ago2, a core component of the RNA-induced silencing complex (RISC) that is required for miRNAs-mediated gene silencing, and potential targets of miRNAs can be isolated from this complex after Ago2 co-immunoprecipitation. Consistently, results of RIP assays confirmed that miR-367 was a target of CASC2 in HCC cells (*P* < 0.01, respectively, Additional file 2: Figure S2A and 2B). Subsequently, the biotin-labeled pulldown system was applied to further confirm that CASC2 could directly interact with miR-367. We observed a significant amount of CASC2 and miR-367 in the CASC2 pulled down pellet compared with control group as measured by real-time PCR (*P* < 0.01, respectively, Additional file 2: Figure S2C and 2D). Furthermore, our data revealed that CASC2 could negatively regulate the expression of miR-367 in HCC cells (*P* < 0.01 and *P* < 0.001, respectively, Fig. 4g). On the other hand, CASC2 expression was significantly elevated or decreased by modulating miR-367 level in HCC cells (*P* < 0.01, respectively, Fig. 4h). Taken together, these data demonstrated that CASC2 could directly bind to miR-367 in HCC cells, and showed a reciprocal repression of CASC2 and miR-367.

### MiR-367 promotes migration, invasion and EMT of HCC cells

**Fig. 5:**
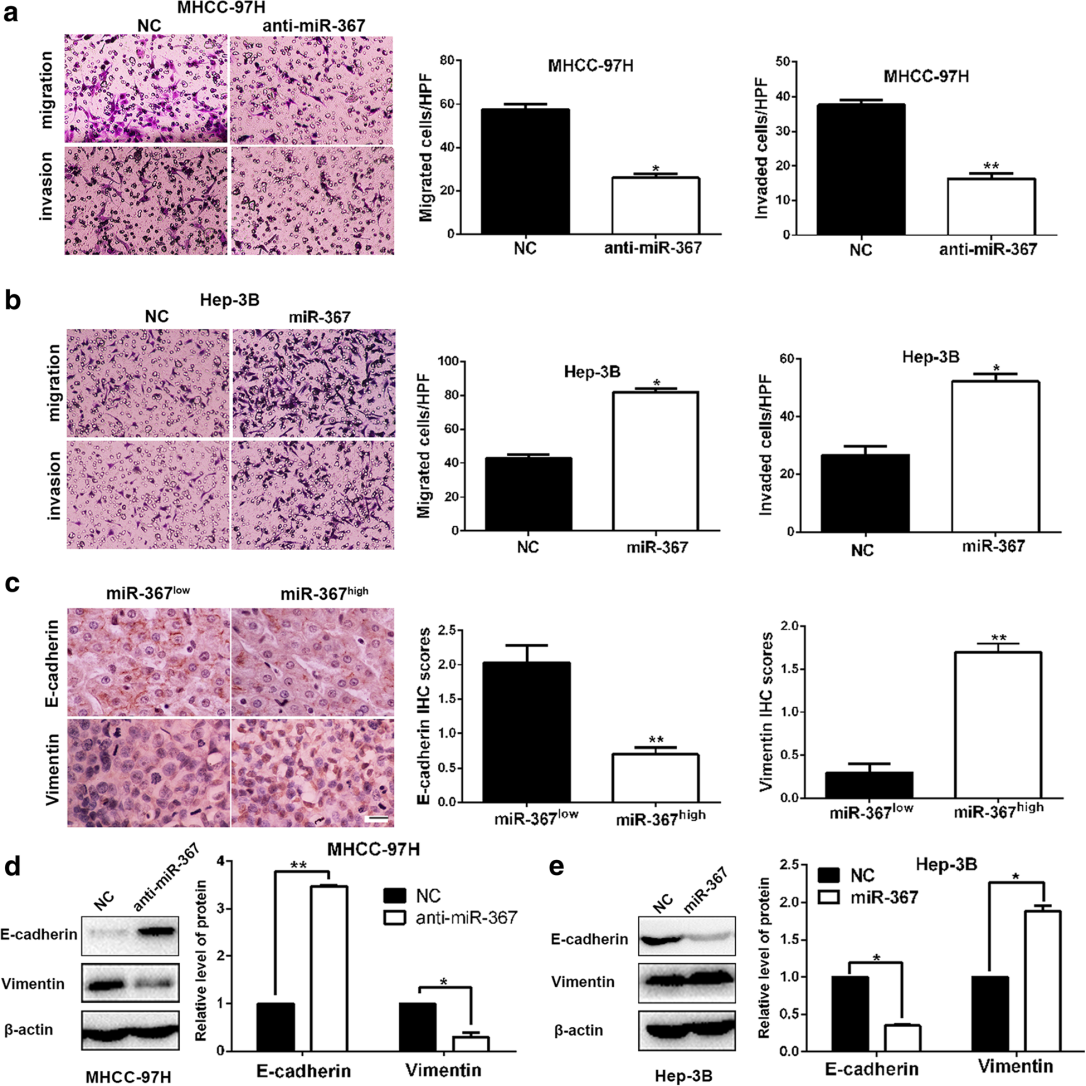
miR-367 promoted migration, invasion and EMT process of HCC cells. **a** miR-367 silencing restrained migration and invasion of MHCC-97H cells. **b** The number migrated and invaded Hep-3B cells were increased after miR-367 restoration. **c** Immunohistochemistry analysis (magnification: ×400; Scale bar:100 μm) showed that HCC tissues in high miR-367 group presented lower E-cadherin expression and higher Vimentin expression compared to those in low miR-367 group. **d** miR-367 knockdown significantly increase E-cadherin expression and decrease Vimentin expression in MHCC-97H cells. **e** miR-367 overexpression facilitated EMT progression of Hep-3B cells. *P < 0.05, **P < 0.01

Next, we were aimed to confirm the roles of miR-367 in migration and invasion of HCC cells. And the results revealed that the migration and invasion abilities of HCC cells were markedly weakened by miR-367 silencing, and significantly enhanced by miR-367 restoration (*P* < 0.05, respectively, Fig. 5a and b). Next, immunohistochemical staining was conducted in 75 HCC samples to explore the associations between miR-367 and EMT markers (Vimentin and E-cadherin). Subgroups (miR-367 low/high) were divided according to the cutoff values of miR-367, which were defined as the median of the cohort. The results indicated that E-cadherin expression was significantly decreased while Vimentin expression was obviously increased in high miR-367 group compared to low miR-367 group (*P* < 0.01, respectively, Fig. 5c). Additionally, Western blot analysis revealed that miR-367 silencing increased E-cadherin expression and decreased Vimentin in MHCC-97H cells (*P* < 0.05, respectively, Fig. 5d). miR-367 overexpression facilitated the EMT process of Hep-3B cells (*P* < 0.05, respectively, Fig. 5e). Therefore, we concluded that miR-367 could promote migration, invasion and EMT of HCC cells.

### MiR-367 directly targeted FBXW7 in HCC cells

**Fig. 6:**
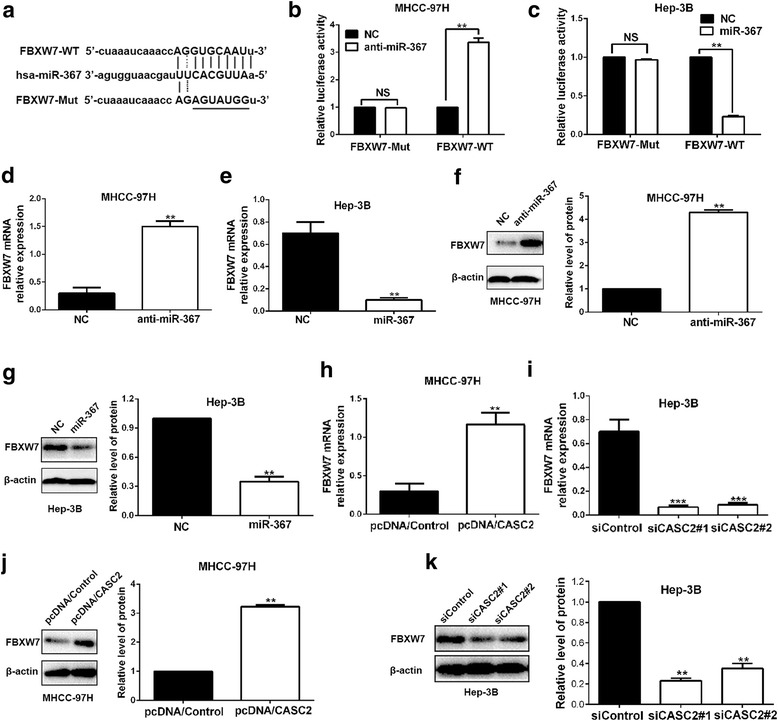
FBXW7 was a target of miR-367 and positively regulated by CASC2 in HCC cells. **a** Bioinformatics analysis showed that CASC2 could directly target 3′-UTR of FBXW7-wild type (WT). FBXW7-mutant (Mut) means mutation of binding sites in the 3′-UTR of FBXW7. **b** and **c** miR-367 negatively regulated the luciferase activity of FBXW7-WT, rather than FBXW7-Mut in HCC cells. **d** and **e** miR-367 negatively regulated the level of FBXW7 mRNA in HCC cells. **f** and **g)** miR-367 inversely regulated the expression of FBXW7 protein in HCC cells. **h** and **i** CASC2 positively regulated the level of FBXW7 mRNA in HCC cells. **j** and **k** CASC2 positively regulate FBXW7 abundance in both MHCC-97H and Hep-3B cells. **P < 0.01, ***P < 0.001

To elucidate the underlying molecular mechanism about how miR-367 exerted its effects on HCC cells, we searched for candidate targets of miR-367 by using bioinformatics tools (microRNA.org and miRBase). As shown in Fig. 6a, binding sequences of miR-367 were identified in 3’UTR of FBXW7 mRNA. Previous study in non-small cell lung cancer has reported that FBXW7 is a downstream target of miR-367 [14]. Besides, it has been confirmed that FBXW7 is a tumor suppressor and suppresses EMT in HCC cells [15, 17]. Moreover, FBXW7 could inhibit the EMT by down-regulating the RhoA signaling pathway in gastric cancer [18]. Then we focused on FBXW7 and assumed that miR-367 could directly target FBXW7 in HCC. Based on our previous studies about FBXW7 [17, 19]. Subsequently, the results of dual luciferase reporter assays, real-time PCR and western blot analysis revealed that miR-367 could directly target FBXW7 and negatively modulate the expression of FBXW7 in HCC cells (*P* < 0.01, respectively, Fig. 6b-g). Next, we explored whether CASC2 could regulate the expression of FBXW7. Our data showed that FBXW7 expression could be positively regulated by CASC2 in HCC cells (*P* < 0.01, respectively, Fig. 6h-k). Thus, we concluded that FBXW7 was a direct target of miR-367 and positively modulated by CASC2 in HCC cells.

### MiR-367/FBXW7 mediates the suppressive roles of CASC2 in migration, invasion and EMT of HCC cells

**Fig. 7:**
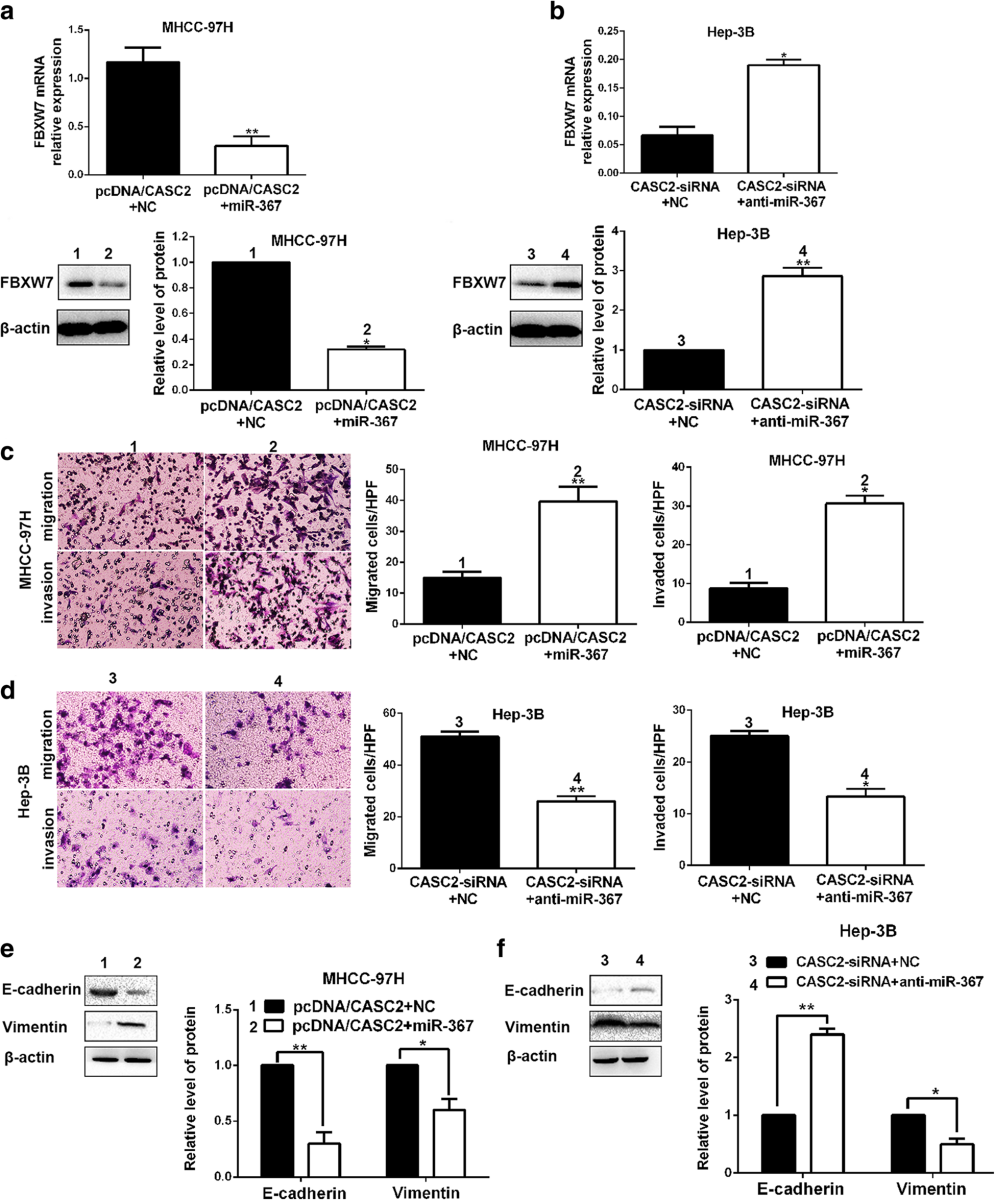
miR-367 mediated the tumor suppressive effects of CASC2 in HCC. **a** miR-367 restoration reduced the expression of FBXW7 in CASC2-overexpressing MHCC-97H cells. **b** miR-367 knockdown increased the level of FBXW7 in CASC2-underexpressing Hep-3B cells. **c** Transwell assays showed that miR-367 could largely reverse the suppressive effects of CASC2 on migration and invasion of MHCC-97H cells. **d** miR-367 silencing abrogated the pro-metastatic effects of CASC2 knockdown in Hep-3B cells. **e** miR-367 could largely reverse the suppressive effects of CASC2 on EMT progression of MHCC-97H cells. **f** miR-367 silencing abolished the promoting effects of CASC2 on EMT progression of Hep-3B cells. *P < 0.05, **P < 0.01

Next, we tried to investigate whether CASC2 could exert its inhibitory effects on migration, invasion and EMT progression of HCC cells through CASC2/miR-367/FBXW7 axis. Notably, miR-367 overexpression reduced the expression of FBXW7 in CASC2-overexpressing MHCC-97H cells and miR-367 knockdown abolished the inhibitory effects of CASC2 silencing on FBXW7 expression in Hep-3B cells (*P* < 0.01, respectively, Fig. 7a and b, Additional file 3: Figure S3A). miR-367 expression was significantly reversed in cells co-transfected with pcDNA/CASC2 and miR-367 mimics compared to pcDNA/CASC2 transfection alone (*P* < 0.01, Additional file 3: Figure S3B). Functionally, Transwell assays indicated that miR-367 overexpression promoted migration and invasion of CASC2-overexpressing MHCC-97H cells (*P* < 0.05, respectively, Fig. 7c, Additional file 3: Figure S3C). Furthermore, the pro-metastatic effects of CASC2 silencing on migration and invasion of Hep-3B cells could be dramatically reversed by miR-367 knockdown (*P* < 0.05, respectively, Fig. 7d). Furthermore, the data from Western blotting indicated that miR-367 could also notably reverse the inhibitory effects of CASC2 on EMT progression of HCC cells (*P* < 0.05, respectively, Fig. 7e and f, Additional file 3: Fig. S3D). Otherwise, FBXW7 overexpression restrained EMT process of Hep-3B cells (Additional file 4: Figure S4A). FBXW7 restoration abrogated promoting effects of miR-367 on migration, invasion and EMT progression of Hep-3B cells (*P* < 0.01, respectively, Additional file 4: Fig. S4B and 4C). Thus, we demonstrated that CASC2 could exert its inhibitory effects of HCC metastasis via CASC2/miR-367/ FBXW7 axis.

### Clinical significance of CASC2 and miR-367 for HCC patients

**Fig. 8:**
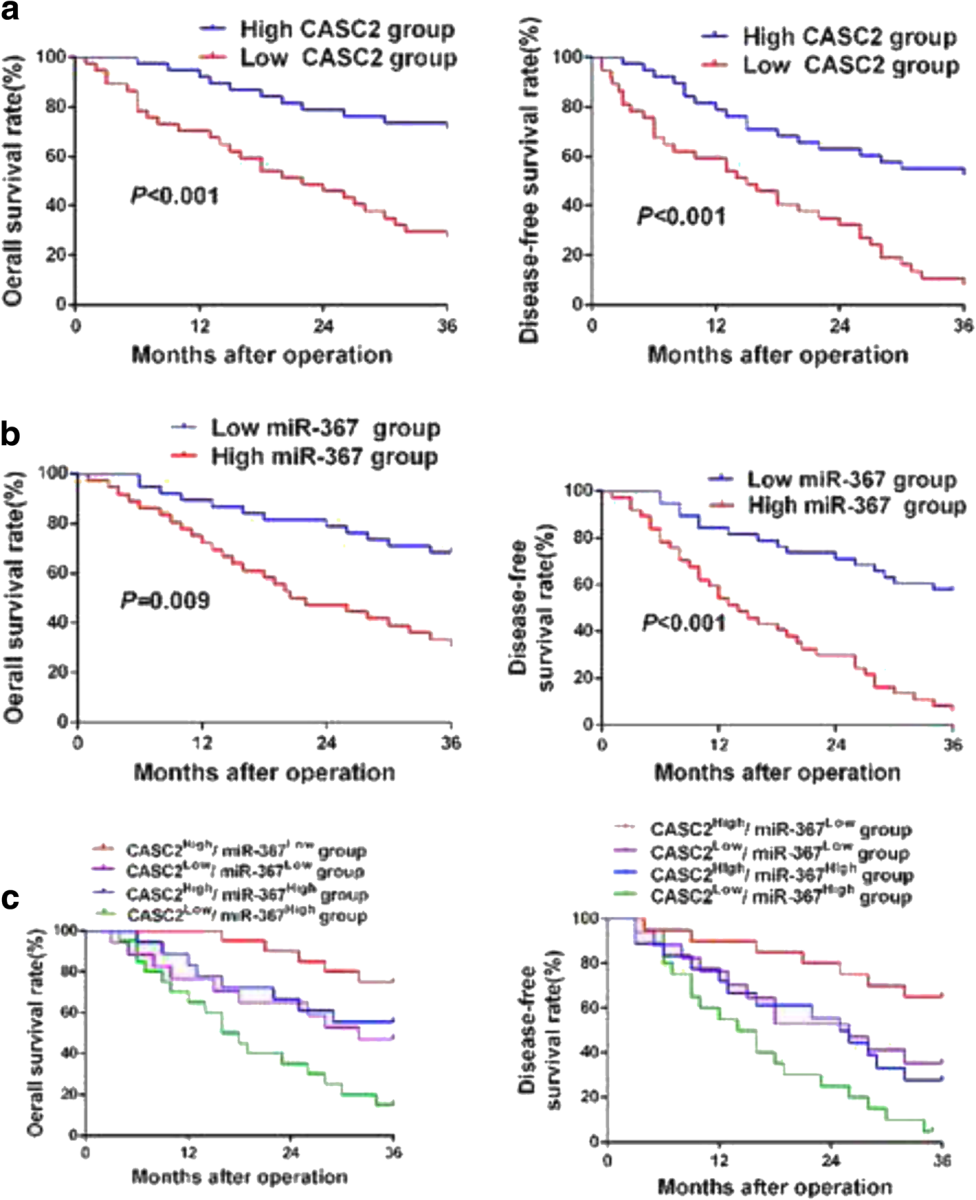
The prognostic significance of CASC2 and miR-367 expression in HCC patients. **a** CASC2 low-expressing HCC patients showed an obvious reduced overall survival (OS) and disease free survival (DFS) compared to CASC2 high-expressing cases. **b** miR-367 high-expressing HCC patients showed an obvious reduced OS and DFS compared to miR-367 low-expressing cases. **c** Patients in CASC2 low and miR-367 high group had the longest OS and DFS, while those in CASC2 high and miR-367 low group showed the shortest OS and DFS. For each cohort, different subgroups were plotted according to the cutoff values of CASC2 and miR-367, which were defined as the median of the cohort

For each cohort, different subgroups were plotted according to the cutoff values of CASC2 and miR-367, which were defined as the median of the cohort. As shown in Table 1, the statistical results indicated that CASC2 underexpression was dramatically associated with venous infiltration (*P* = 0.004), high Edmondson-Steiner grading (*P* = 0.043) and advanced TNM tumor stage (*P* = 0.003). Meanwhile, miR-367 overexpression was notably correlated with large tumor size (*P* = 0.015), venous infiltration (*P* = 0.025) and advanced TNM tumor stage (*P* = 0.020). Therefore, we suggested that aberrant expression of CASC2 and miR-367 were closely correlated with the metastasis-associated clinicopathologic features of HCC. Subsequently, 3-year overall survival (OS) and disease-free survival (DFS) were analyzed by Kaplan–Meier survival curves. We found that HCC patients in low CASC2 group presented a significant poorer outcome compared to those in high CASC2 group (*P* < 0.001, respectively. Fig. 8a). Meanwhile, miR-367 high-expressing HCC patients showed a shorter OS and DFS compared to miR-367 low-expressing cases. (*P* < 0.01, respectively. Fig. 8b). Among these four subgroups, HCC patients with high CASC2 expression and low miR-367 expression had the best prognosis, while CASC2 low-expressing and miR-367 high-expressing HCC patients showed the poorest prognosis (Fig. 8c). These data indicated that CASC2 and miR-367 expression, especially their combination, may be ponderable and promising factors for predicting the prognosis of HCC patients.

**Table 1:** Correlation between the clinicopathologic characteristics and CASC2 and miR-367 expression in HCC (*n* = 75)

Clinical parameters	Cases	Expression level		*P* value	Expression level		*P* value
		CASC2^high^ (*n* = 38)	CASC2^low^ (*n* = 37)		miR-367^high^ (*n* = 38)	miR-367^low^ (*n* = 37)	
Age(years)							
< 50	24	13	11	0.677	10	14	0.285
≥ 50	51	25	26		28	23	
Gender							
Male	64	34	30	0.304	32	32	0.781
Female	11	4	7		6	5	
HBV							
Absent	13	9	4	0.141	7	6	0.801
Present	62	29	33		31	31	
Serum AFP level (ng/mL)							
< 400	18	11	7	0.127	6	12	0.092
≥ 400	57	27	30		32	25	
Tumor size (cm)							
< 5	34	21	13	0.080	12	22	0.015^a^
≥ 5	41	17	24		26	15	
Number of tumor nodules							
1	62	35	28	0.104	30	32	0.389
≥ 2	13	4	9		8	5	
Cirrhosis							
Absent	20	12	8	0.330	7	13	0.102
Present	55	26	29		31	24	
Venous infiltration							
Absent	54	33	21	0.004^a^	23	31	0.025^a^
Present	21	5	16		15	6	
Edmondson-Steiner grading							
I + II	49	29	20	0.043^a^	22	27	0.170
III + IV	26	9	17		16	10	
TNM tumor stage							
I + II	56	34	22	0.003^a^	24	32	0.020^a^
III + IV	19	4	15		14	5	

*HCC* hepatocellular carcinoma, *HBV* hepatitis B virus, *AFP* alpha-fetoprotein, *TNM* tumor-node-metastasis

^a^Statistically significant

## Discussion

LncRNAs, that function as novel diagnostic biomarkers, have intimate connection with the progression of HCC [20]. For instance, it has been reported that HCC development is accelerated by lncRNA CCAT1 via acting as let-7 sponge [21]. Accordingly, CASC2 has been identified as a robust tumor suppressor in several cancers [12]. In the present study, we found that CASC2 expression was markedly suppressed in HCC tissues and cells. Moreover, the expression of CASC2 was negatively associated with the aggressiveness and recurrence of HCC. Consistently, the data analysis from R2: Genomics Analysis and Visualization Platform (http://r2.amc.nl) including GEO and TCGA database showed that CASC2 was significantly underexpressed in HCC tissues. Thus, we proposed that CASC2 might be a tumor suppressor in HCC.

Migration and invasion abilities of cancer cells are closely related to the aggressiveness and recurrence of HCC [2, 22, 23]. And more and more lncRNAs have been identified to be regulators of migration and invasion of HCC cells [24, 25]. Here, we found that CASC2 could restrain the migration and invasion abilities of HCC cells both in vitro and in vivo. Furthermore, CASC2 could inhibit the EMT progression of HCC cells. Thus, we concluded that CASC2 functioned as a tumor suppressor by suppressing migration, invasion and EMT progression of HCC cells.

It has been reported that the abnormally expressed lncRNAs act as ceRNAs for miRNAs to modulate tumor development [26]. In this study, we found that miR-367 expression was obviously upregulated and negatively correlated with CASC2 in HCC tissues. Besides, bioinformatics analysis, luciferase reporter assay, biotin pull-down assay and RIP assay defined that miR-367 was a target of CASC2 in HCC cells. And a reciprocal repression of CASC2 and miR-367 was existed in HCC cells. Previous study reported that miR-367 promoted proliferation, migration and invasion of HCC cells [13]. Thus, we speculated that CASC2 exerted its suppressive effects on HCC cells via interacting with miR-367. The results from loss- and gain-of-function experiments presented that miR-367 could promote migration, invasion and EMT processes of HCC cells. Then bioinformatics tools were used to identify the potential downstream targets of miR-367. The analysis suggested that FBXW7 might be a downstream target of miR-367. In our previous studies, FBXW7 has been confirmed to be an tumor suppressor in HCC [17, 19, 27]. Besides, FBXW7 had been confirmed as a target of miR-367 in non-small cell lung cancer, and could suppress EMT progression of HCC cells [14, 15]. Moreover, previous studies suggested that FBXW7 suppressed EMT of tumor cells by targeting c-Myc [28], Notch [29], mTOR [30, 31] and RhoA signaling pathway [18]. In this study, we consistently found that FBXW7 could suppress EMT of HCC cells. Further studies are worth to be performed to investigate the underlying mechanisms involved in FBXW7 regulation of EMT in HCC. Subsequently, we explored that CASC2 could positively regulate the expression of FBXW7 via targeting miR-367 in HCC cells. Moreover, miR-367 mediated the anti-metastatic role of CASC2 in HCC cells. In accordance, FBXW7 restoration abolished the promoting effects of miR-367 on migration, invasion and EMT progression of HCC cells. In all, these results demonstrated that CASC2 could restrain cell migration, invasion and EMT process via CASC2/miR-367/ FBXW7 axis in HCC.

Previous studies have found that low expression of CASC2 and high level of miR-367 expression correlated with poor prognosis of lung cancer and pancreatic cancer [32, 33]. Here, CASC2 underexpression and miR-367 overexpression were closely correlated with the metastasis-associated clinicopathologic features, such as venous infiltration, high Edmondson-Steiner grading and advanced TNM tumor stage. HCC patients with low CASC2 expression or high miR-367 expression had a obvious poorer prognosis. Besides, CASC2 high-expression and miR-367 low-expressing HCC patients showed the best prognosis, while HCC patients with low CASC2 expression and high miR-367 expression had the poorest prognosis. Thus, we concluded that CASC2 and miR-367 expression, especially their combination, could be ponderable and promising factors for predicting the prognosis of HCC patients.

In conclusion, we demonstrated that CASC2 was downregulated in HCC, and could inhibit migration, invasion and EMT progression of HCC cells via CASC2/miR-367/FBXW7 axis, which could be a valuable and promising therapeutic target for HCC in the future.

## Conclusions

Our study reported an observation that CASC2 underexpression, miR-367 overexpression and their combination could lead to poor prognosis of HCC patients. Next, We found that CASC2 inhibited migration and invasion of HCC cells in vitro and in vivo. Furthermore, CASC2 inversely modulated EMT process of HCC cells. miR-367 was identified as not only a target but also a functional mediator of CASC2 in HCC cells. FBXW7 suppressed migration, invasion and EMT progression of HCC cells and directly targeted by miR-367. To conclude, CASC2/miR-367/FBXW7 axis suppressed EMT and mobility of HCC cells. This finding will improve understanding of mechanism involved in cancer progression and provide novel targets for the molecular treatment of HCC.

## Additional files

Additional file 1: Figure S1. The expression of CASC2 in a public database. (**A**) TCGA data from R2: Genomics Analysis and Visualization Platform (http://r2.amc.nl) showed that CASC2 expression was down-regulated in HCC tissues compared to normal liver tissues. (**B**) GEO data (GSE45436) from R2: Genomics Analysis and Visualization Platform (http://r2.amc.nl) indicated that the expression of CASC2 was obviously lower than that in normal liver tissues. (TIFF 271 kb)

Additional file 2: Figure S2. RIP assay revealed that miR-367 was a target of CASC2. (**A**) The association between CASC2, miR-367 and Ago2 was ascertained by analyzing Hep-3B and MHCC-97 L cell lysates using RNA immunoprecipitation with an Ago2 antibody. (**B**) Real-time PCR was used to detect the CASC2 level change in the substrate of RIP assay in miR-367-overexpressing HCC cells. (**C**) Detection of CASC2 using real-time PCR in the sample pulled down by biotinylated CASC2 and negative control (NC) probe. (**D**) Detection of miR-367 using real-time PCR in the same sample pulled down by biotinylated CASC2 and NC probe. Input was used for normalization. **P < 0.01, ***P < 0.001. (TIFF 283 kb)

Additional file 3: Figure S3. miR-367 reversed the anti-metastatic effects of CASC2 on HCC cells. (**A**) FBXW7 level was reversed by miR-367 in CASC2-overexpressing MHCC-97H cells. (**B**) miR-367 expression was rescued by miR-367 mimics in CASC2-overexpressing MHCC-97H cells. (**C**) miR-367 abolished the inhibitory effects of CASC2 on migration and invasion of MHCC-97H cells. (**D**) miR-367 abrogated the inhibitory effects of CASC2 on EMT progression of MHCC-97H cells. *P < 0.05, **P < 0.01, ***P < 0.001. (TIFF 1008 kb)

Additional file 4: Figure S4. FBXW7 rescued the pro-metastatic effects of miR-367 on HCC cells. (**A**) FBXW7 overexpression increased the expression of E-cadherin and decreased the expression of Vimentin in Hep-3B cells. (**B**) FBXW7 abolished the promoting effects of miR-367 on EMT progression of Hep-3B cells. (**C**) FBXW7 abrogated the pro-metastatic effects of miR-367 on migration and invasion of Hep-3B cells. **P < 0.01. (TIFF 878 kb)

## Abbreviations

3'-UTR: 3'-untranslated region
AFP: Alpha-fetoprotein
CASC2: Cancer susceptibility candidate 2
EMT: Epithelial-mesenchymal transition
FBXW7: F-box and WD repeat domain containing 7
H&E: Hematoxylin and eosin
HBV: Hepatitis B virus
HCC: Hepatocellular carcinoma
IHC: Immunohistochemistry
lncRNA: Long non-coding RNA
miRNA: microRNA
RIP: RNA immunoprecipitation
TNM: Tumor-node-metastasis
