# Supplementary figures and images for: Long non-coding RNA CASC2 suppresses epithelial-mesenchymal transition of hepatocellular carcinoma cells through CASC2/miR-367/FBXW7 axis
Source: Mol Cancer. 2017 Jul 17;16:123. doi: 10.1186/s12943-017-0702-z (PMC5514467; doi:10.1186/s12943-017-0702-z)

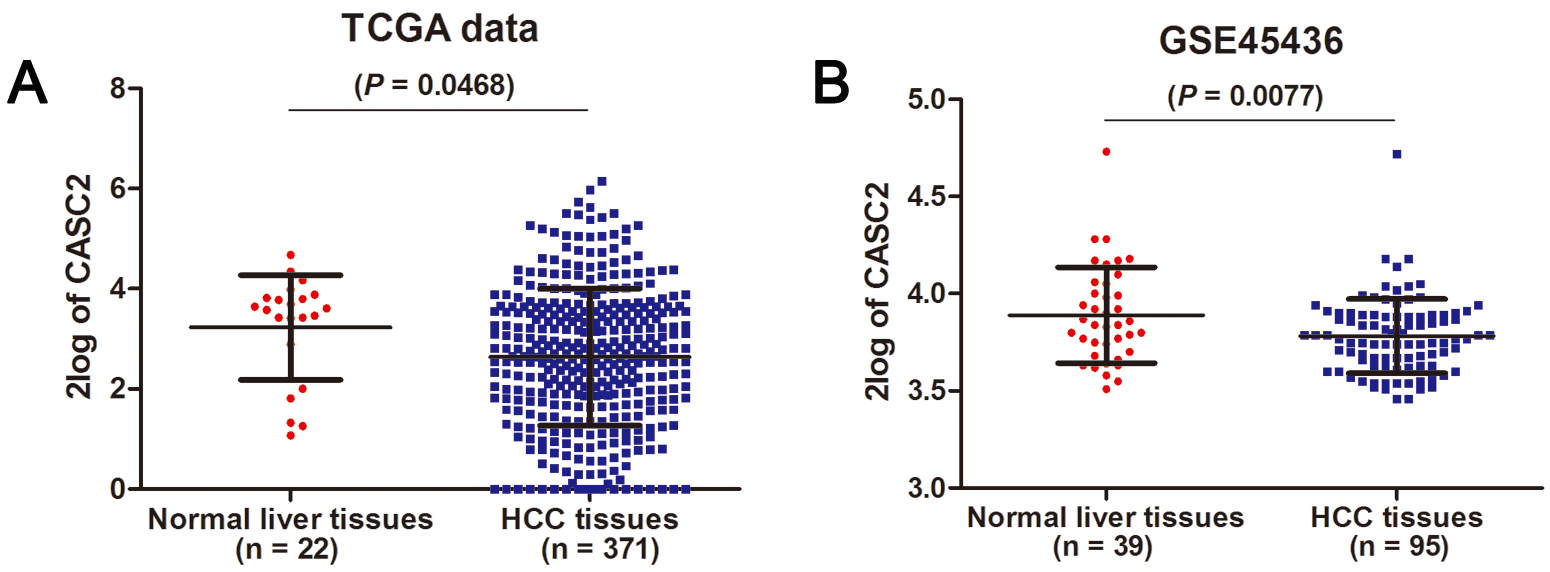

Supplement: Supplementary file 1 — The expression of CASC2 in a public database. (A) TCGA data from R2: Genomics Analysis and Visualization Platform (http://r2.amc.nl) showed that CASC2 expression was down-regulated in HCC tissues compared to normal liver tissues. (B) GEO data (GSE45436) from R2: Genomics Analysis and Visualization Platform (http://r2.amc.nl) indicated that the expression of CASC2 was obviously lower than that in normal liver tissues. (TIFF 271 kb) [file 12943_2017_702_MOESM1_ESM.tif]

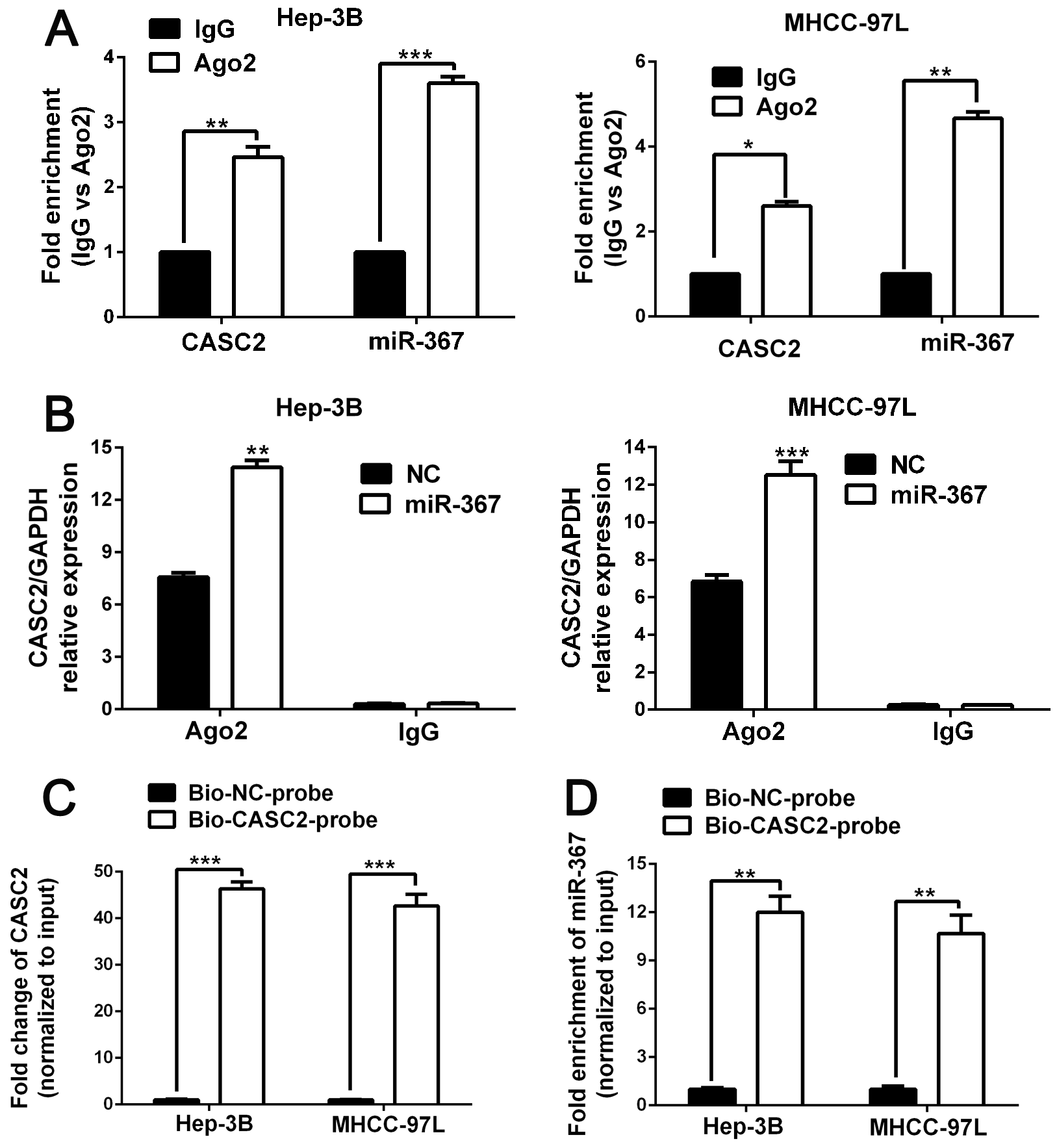

Supplement: Supplementary file 2 — RIP assay revealed that miR-367 was a target of CASC2. (A) The association between CASC2, miR-367 and Ago2 was ascertained by analyzing Hep-3B and MHCC-97 L cell lysates using RNA immunoprecipitation with an Ago2 antibody. (B) Real-time PCR was used to detect the CASC2 level change in the substrate of RIP assay in miR-367-overexpressing HCC cells. (C) Detection of CASC2 using real-time PCR in the sample pulled down by biotinylated CASC2 and negative control (NC) probe. (D) Detection of miR-367 using real-time PCR in the same sample pulled down by biotinylated CASC2 and NC probe. Input was used for normalization. **P < 0.01, ***P < 0.001. (TIFF 283 kb) [file 12943_2017_702_MOESM2_ESM.tif]

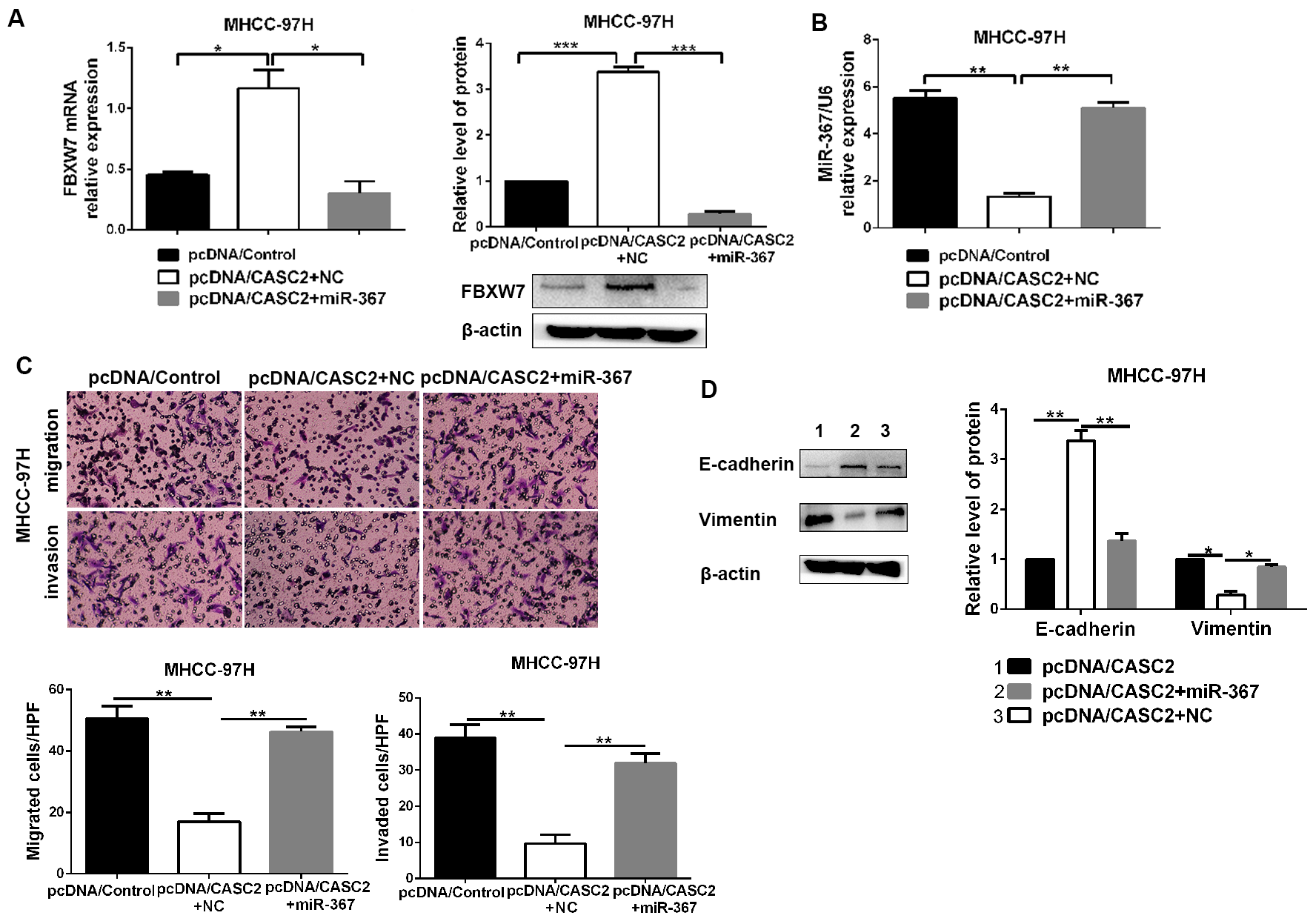

Supplement: Supplementary file 3 — miR-367 reversed the anti-metastatic effects of CASC2 on HCC cells. (A) FBXW7 level was reversed by miR-367 in CASC2-overexpressing MHCC-97H cells. (B) miR-367 expression was rescued by miR-367 mimics in CASC2-overexpressing MHCC-97H cells. (C) miR-367 abolished the inhibitory effects of CASC2 on migration and invasion of MHCC-97H cells. (D) miR-367 abrogated the inhibitory effects of CASC2 on EMT progression of MHCC-97H cells. *P < 0.05, **P < 0.01, ***P < 0.001. (TIFF 1008 kb) [file 12943_2017_702_MOESM3_ESM.tif]

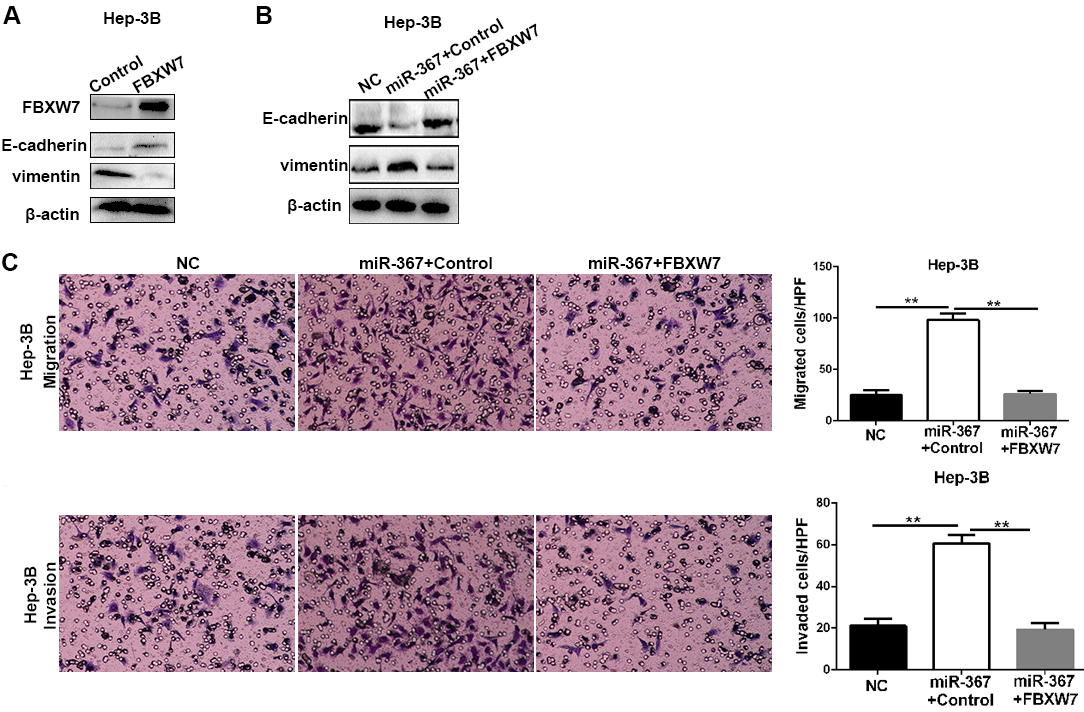

Supplement: Supplementary file 4 — FBXW7 rescued the pro-metastatic effects of miR-367 on HCC cells. (A) FBXW7 overexpression increased the expression of E-cadherin and decreased the expression of Vimentin in Hep-3B cells. (B) FBXW7 abolished the promoting effects of miR-367 on EMT progression of Hep-3B cells. (C) FBXW7 abrogated the pro-metastatic effects of miR-367 on migration and invasion of Hep-3B cells. **P < 0.01. (TIFF 878 kb) [file 12943_2017_702_MOESM4_ESM.tif]
